# Parental positive affect and negative affect in same- and different-sex parent families: no associations with parental gender and caregiving role

**DOI:** 10.3389/fpsyg.2024.1332758

**Published:** 2024-03-07

**Authors:** Tamara L. M. Leter, Kate Ellis-Davies, Bérengère Rubio, Olivier Vecho, Henny M. W. Bos, Michael E. Lamb, Loes Van Rijn – Van Gelderen

**Affiliations:** ^1^Research Institute of Child Development and Education, University of Amsterdam, Amsterdam, Netherlands; ^2^Department of Psychology, College of Human and Health Sciences, Swansea University, Swansea, United Kingdom; ^3^Département de psychologie, Université Paris Nanterre, UR CLIPSYD, Nanterre, France; ^4^Department of Psychology, Faculty of Biology, School of Biological Sciences, University of Cambridge, Cambridge, United Kingdom

**Keywords:** positive affect, negative affect, fathers, mothers, primary caregivers, secondary caregivers, parent–child observations

## Abstract

Positive and negative parental affect influence developing parent–child attachment relationships, especially during infancy as well as children’s social–emotional, academic, and behavioral functioning later in life. Increasingly, because both mothers and fathers can play central caregiving roles, the parenting qualities of both parents demand consideration. Therefore, this study investigated whether parental gender and caregiving role were associated with mothers’ and fathers’ positive affect and negative affect during interactions with their 4-month-old firstborn infant, while determining whether parenting stress, infant temperament, having a singleton/twin, and living in the Netherlands, France, or the United Kingdom were related to parental positive affect and negative affect. In all, 135 different-sex, same-sex male, and same-sex female couples (113 fathers and 157 mothers, comprising 147 primary, and 123 secondary caregivers) who conceived through artificial reproductive techniques were studied. The couples were videorecorded at home while in feeding, cleaning, and playing contexts to assess the levels of positive and negative parental affect. In addition, the couples completed questionnaires about their caregiving role, parenting stress, and the infants’ temperament. Mixed linear models indicated that the levels of positive and negative parental affect toward the infant in all contexts were not related to parental gender, caregiving role, the interaction between parental gender and caregiving role, parenting stress, infant temperament, or singleton/twin status. However, the target parental behaviors were related to the country of origin, suggesting differences among Dutch, French, and British parents. Overall, we found no evidence that gender or caregiving roles were associated with the levels of positive and negative affect shown by the parents.

## Introduction

Fathers are increasingly involved in the family (Cabrera et al., 2000; Yeung et al., 2001), and increased access to parental leave for fathers is enhancing paternal involvement (Huerta et al., 2014). However, mothers are still less likely to be employed after the birth of their children and tend to spend more time caring for their children than fathers (Endendijk et al., 2018), perhaps in response to societal expectations and gender stereotypes, and this affects how parents behave toward their children (Endendijk et al., 2017, 2018). Gender-dependent qualities may increase the likelihood that mothers and fathers will treat their children differently (Popenoe, 1996; Cabrera et al., 2000).

However, most research on gender differences in parenting has involved traditional families, with male and female biological parents, and more importantly, with mothers as the parents most concerned with caregiving responsibilities (i.e., primary caregivers) and fathers as secondary caregivers (Rubio et al., 2017). With primary caregiving mothers being the focus of most research on parenting, it is unclear whether behavioral differences between mothers and fathers are attributable to gender or to caregiving role. For example, Abraham et al. (2014) found that primary caregivers, regardless of the parents’ gender, showed similar patterns of activity in their ‘parenting caregiving’ neural networks (e.g., amygdala), at levels greater than in secondary caregiving fathers. This suggests the importance of caregiving role. However, caregiving role is seldom considered in research on mothers’ and fathers’ parenting quality. This study explored the relative importance of gender and caregiving role in shaping differences between mothers’ and fathers’ levels of infant-directed positive and negative affect.

### Positive and negative affect

Whereas 4– to 5-month-old infants cannot understand their parents’ language, they can recognize emotional expressions and to some degree the valence of parental speech (Bornstein, 2012). Parents can display such expressions by showing positive or negative affect toward their infants. Positive affect involves parents showing pleasure when interacting with their infants by smiling with eye contact, laughing, warm intonation in the voice, and physically touching or displaying affection (for example, by hugging), and speaking with a warm intonation (Landry et al., 2008; Lunkenheimer et al., 2011; Kwon et al., 2012). Negative affect involves parents expressing irritability, anger, or hostility when interacting with their infants though negative tone, raised voice, negative comments, negative facial expressions (e.g., frowning or eye rolling), or sighing (Morris et al., 2002; Lunkenheimer et al., 2011; Kwon et al., 2012). According to Bowlby, children develop mental representations of attachment figures depending on how those figures treat them (Bowlby, 1969; Atzaba-Poria and Pike, 2015). These mental representations shape children’s thoughts and behavior about themselves and relationships with others (Bowlby, 1969). Positive and negative affect also relate to parents’ emotional availability, which provides feedback on how the parents perceive the child (Biringen et al., 2000). Furthermore, the expression of appropriate emotions may play a key role in effective parenting by activating, engaging, and regulating positive interactions with children (Dix, 1991). At the same time, when parents experience emotions that are too strong, this can undermine rather than enhance effective parenting by, for example, leading them to express negative emotions instead of behaving in a pedagogically effective way (Dix, 1991), with less optimal child outcomes as a consequence.

Empirical studies have supported these theoretical ideas regarding the influence of parental positive and negative affect on children’s social–emotional, academic, and behavioral functioning later in life. For example, a meta-analysis showed that more parental warmth and affection were related to better psychological adjustment in school-aged children (Khaleque, 2013). Studies focused on younger children also found that positive affect matters. One study showed that parents who directed more positive affect to their 3-month-old infants had infants who were more likely to be securely attached to them as 1-year-olds (Cox et al., 1992), while another study showed that 3-to 4-year-old boys whose parents expressed more positive affect were better accepted by peers 1 year later (Pali et al., 2022). On the other hand, research shows that negative parental affect has adverse effects on children’s social–emotional, academic, and behavioral functioning (Taraban and Shaw, 2018) and is an early childhood risk factor for the development of externalizing and internalizing problems in children and adolescents (Alemany et al., 2013). A systematic review by Samdan et al. (2020) indicated that negative parenting, defined as harsh parenting and hostility, is associated with infants’ excessive crying and problematic eating behavior. Thus, both forms of affect are relevant to children’s development, but do fathers and mothers of young infants display similar affect?

For a long time, it was assumed that gender was one of the factors affecting the amount of affect expressed by parents, with women considered more emotional and emotionally expressive than men (Grossman and Wood, 1993). Some research also suggests that women are more aware of and knowledgeable about emotions than men (Barrett et al., 2000) and are more capable of labeling facial expressions (Montagne et al., 2005). We might, therefore, expect mothers to express more positive and negative affect than fathers toward their infants.

Examining positive affect, Brundin et al. (1988) showed that mothers of 6-month-olds laughed and vocalized more than fathers did. Similarly, more positive affect was expressed in mother-toddler than father-toddler interactions (Lunkenheimer et al., 2011) in both dyadic and triadic contexts (Kwon et al., 2012). However, few researchers have explored the differences between mothers’ and fathers’ negative affect. Research with 1-to 11-year-old children and their parents found that mothers reported expressing more negative affect than fathers, but only toward the youngest child when parents had multiple children (Deater-Deckard, 1996). Other observational research found that, in both dyadic and triadic contexts, mothers showed less negative affect toward their toddlers than fathers did (Kwon et al., 2012). However, those studies of gender differences in parental positive and negative affect have not considered the possible effect of the caregiving role.

Examining positive affect, gender, and caregiver role, a study of 3-to 6-month-old infants and their different-sex parents found that mothers directed more positive affect toward their infants than fathers did, regardless of the parents’ employment status (Forbes et al., 2004). However, employment status is not necessarily indicative of caregiving role because a full-time working parent can still be the primary caregiver. Observational research with 1-year-old infants and their different-sex parents found that mothers were more involved in caregiving and displayed more affection, vocalizations and smiling than fathers (Sun and Roopnarine, 1996). This was also true for older children, showing that mothers remained the primary caregivers of their 32-to-72-months old children, even when mothers were employed, with mothers displaying more positive affect than fathers (Stuckey et al., 1982). These studies all showed that primary caregiving mothers showed more positive affect than secondary caregiving fathers. The studies did not investigate differences between primary and secondary caregivers of both genders.

Earlier studies ostensibly controlling for caregiving role showed that differences in positive affect were related to parental gender. An observational study of 8-month-old firstborn infants and their parents indicated that mothers showed more affectionate and touching behavior, vocalizations, smiling, and attention to their children than fathers did regardless of the parents’ caregiving roles (Lamb et al., 1982), and when dual-career parents reported an equal division of caregiving tasks (Field et al., 1987). Other research with 8-to-12-month-old infants similarly showed that mothers were more affectionate than fathers regardless of whether the fathers had been primary caregivers (Hwang, 1986). Furthermore, research with 9-and 15-month-old infants and their parents, who both worked full-time, indicated that the mothers were primarily responsible for caregiving, both parents were equally involved in playing, and that mothers vocalized more (a component of positive affect) during play than fathers (Laflamme et al., 2002). Finally, regarding positive affect, Field (1978) reported that primary caregiving fathers and mothers smiled and vocalized more, and imitated facial expressions more than secondary caregiving fathers did.

Differences in positive affect have been related to caregiving role rather than parental gender in some studies. For example, when fathers were observed in one-on-one interaction with their 8-to-12-month-old infants, those who were not primary caregivers showed more affection than those who were (Hwang, 1986).

Examining negative affect, gender, and caregiver role, one study found that highly educated and stressed fathers with demanding jobs were reported by both mothers and fathers to be more irritable with their children than mothers were (Heath, 1976). Other studies found that employed fathers who were highly involved in caregiving expressed more negative affect toward their 4-month-old infants when the mothers worked part-time than when the mothers were unemployed (Grych and Clark, 1999). However, the effect of employment and caregiving role were not properly distinguished, the fathers’ caring roles were unclear, and the mothers’ behavior was not examined, making it impossible to determine whether differences were related to parental gender or caregiving role.

In sum, all empirical studies of differences between mothers and fathers in parental positive and negative affect failed to include secondary caregiver mothers whose inclusion is necessary to fully distinguish between the contributions of parental gender and caregiving role. In addition, most of these studies were conducted decades ago, before major changes in the context of parenting and the accessibility of artificial reproductive techniques that have made it easier for both mothers and fathers to play central caregiving roles. Furthermore, all the research reported above involved parents in different-sex couples, with little to no research on secondary caregiving mothers. Researchers clearly need to compare primary caregiver mothers and fathers, and secondary caregiver mothers and fathers (Carone and Lingiardi, 2022). Artificial reproductive techniques (ART) are increasingly sophisticated and accessible, making it possible for same-sex male and same-sex female parents to have children. Studying the latter parents, as we did in this study, provides a unique opportunity to assess the independent effects of parental gender and caregiving role while controlling for child and parent characteristics that might also influence parenting quality.

Child temperament can significantly shape parent–child interactions (Belsky, 1984), with parents of children with difficult temperaments (negative emotionality) expressing less positive affect and more negative affect toward their children (Taraban and Shaw, 2018). Stress can adversely affect parental wellbeing making parents less tolerant and more irritable with their children (Bornstein, 2012; McFadden and Tamis-Lemonda, 2013), especially those who have difficult temperaments (negative emotionality) (Taraban and Shaw, 2018). In addition, having singletons as opposed to twins can also affect parent–child interaction. Twins demand more care and thus create more stress for parents than singletons do (Lytton and Gallagher, 2012). Because mothers often specialize in nurturing and fathers in play (Lamb, 2010) it is valuable to examine differences in parental behavior in diverse contexts. Lastly, countries differ with respect to views of same-sex parents (Takács et al., 2016), the use of ART (González, 2019), and gender stereotypes. Because previously reported differences between the parents in different countries, notably in parental sensitivity and intrusiveness (Ellis-Davies et al., 2022), have been inconsistent (Ellis-Davies et al., 2022), we were not able to formulate hypotheses about specific national differences we might find in our study. However, we expected that the country of residence would be related to different levels of affect. Therefore, this study of positive and negative parental affect both controlled for and examined the correlates of infant temperament, parenting stress, singleton versus twin status, and country of residence.

### Current study

Given the role of positive and negative affect in both attachment formation (Cox et al., 1992) and social–emotional, academic, and behavioral functioning (Alemany et al., 2013; Taraban and Shaw, 2018; Samdan et al., 2020; Pali et al., 2022), as well as increasing paternal involvement in many countries (Huerta et al., 2014), it is important to study the impact of gender and caregiving role on parents’ expressions of positive and negative affect. We did so by observing mothers’ and fathers’ displays of positive and negative affect while feeding, cleaning, and playing with their 4-month-old first-born infants. As explained earlier, the study also considered parent–child factors (parenting stress and infant temperament) and contextual factors (namely: singleton versus twin status and country of residence).

Based on previous research, we expected mothers to show more positive affect than fathers (Field, 1978; Lamb et al., 1982; Hwang, 1986; Field et al., 1987; Brundin et al., 1988; Sun and Roopnarine, 1996; Laflamme et al., 2002) and that mothers and fathers would display different levels of negative affect as well (Deater-Deckard, 1996; Kwon et al., 2012). There is a lack of prior research on this topic, and we could not predict the effect of the caregiving role on the parents’ positive and negative affect.

## Methods

### Participants

The participants in the current study were part of the New Parents Study (NPS) of collaborating Dutch, British, and French researchers (see also: Rubio et al., 2017; Van Rijn-van Gelderen et al., 2018, 2020; Ellis-Davies et al., 2022). The NPS consisted of 140 two-parent families from the Netherlands (33.6%), the United Kingdom (23.6%), and France (42.8%), 38 of whom were same-sex male parent families, 61 same-sex female parent families, and 41 different-sex parent families. Only families who participated in the video-recorded observations when their children were around 4 months old were included in the analytic sample of this study. Therefore, the sample consisted of 135 two-parent families (*N* = 270 parents) from the Netherlands (34.8%), the United Kingdom (23.0%), and France (42.2%), 36 of whom were same-sex male parent families, 58 same-sex female parent families, and 41 different-sex parent families. For families with twins (*N* = 42), the observations of only one (randomly selected) twin were included in the analyses.

The analytic sample consisted of 113 fathers and 157 mothers (*N* = 270) between 22 and 59 years old (*M* = 35.11, *SD* = 5.36). At the time of the observations, the infants had a mean age of 3.68 months (*SD* = 0.59). Most couples had singletons (85.2%) and girls (60.0%). The duration of the relationship between the parents ranged from 2.00 to 16.50 years (*M* = 8.11, *SD* = 3.60). The majority of the couples were married or registered as partners (80.0%) for an average duration of 3.49 years (*SD* = 3.05), and the others were cohabiting (20.0%), with an average duration of 6.68 years (*SD* = 3.46). The majority (61.7%) of the parents worked full-time, 24.9% of the parents worked part-time, and 13.4% of the parents did not work outside the home. Most parents were highly educated (82.5%), indicating that the parents had a college or higher degree. Families lived in small cities (33.3%), medium cities (31.9%), or large cities (28.9%), with a few in rural areas (5.9%). Most families had annual incomes of more than 42,356 dollars (71.6%), with the remaining families having annual incomes between 12,706 and 42,356 dollars (26.9%) or less than 12,706 dollars (1.5%).

To distinguish which parent was the primary caregiver and which parent was the secondary caregiver, “The Who Does What” questionnaire (Cowan and Cowan, 1990) was used. Both parents answered 6 items, on a scale from 1 (*I do it all*) to 9 (*Partner does it all*), about responsibility for caregiving tasks during the weekdays, namely: (1) when getting up, during breakfast, and when dressing the infant, (2) during the day from 9.00 a.m. to 1.00 p.m., (3) during the day from 1.00 p.m. to 5.00 p.m., (4) when having dinner, during playtime, at bedtime, (5) in the evening until midnight, and (6) when the infant needed care in the middle of the night. Multiple imputation, with *m* = 20 imputations, was used for missing data in 13 cases (for more information see: Ellis-Davies et al., 2022). The questionnaire resulted in a score for both parents, in which the parent with the lowest score was identified as primary caregiver and the other parent as secondary caregiver. In some cases, due to multiple imputation, both parents were identified as primary caregivers (Ellis-Davies et al., 2022). Eventually, 147 parents were identified as primary caregivers and 123 as secondary caregivers. In Table 1 the gender and the family type of primary and secondary caregivers are presented.

**Table 1:** Caregiving role disaggregated by parental gender and family type.

Gender			Primary caregiver	Secondary caregiver	Total
Male	Family type	Same-sex male	40	32	72
		Different sex	5	36	41
	Total		45	68	113
Female	Family type	Same-sex female	63	53	116
		Different sex	39	2	41
	Total		102	55	157
Total	Family type	Same-sex male	40	32	72
		Same-sex female	63	53	116
		Different sex	44	38	82
	Total		147	123	270

In Table 2 the demographic characteristics of mothers and fathers as well as primary and secondary caregivers are compared. Table 2 shows that there were significant differences between mothers and fathers regarding their age, the length of the relationships, marital status, having a twin, working status, educational level, income, and country of residence. There were no significant differences between mothers and fathers regarding their living location. Table 2 also shows the only significant difference between primary and secondary caregivers related to their work status: secondary caregivers more often worked full-time and were less likely to be unemployed than primary caregivers.

**Table 2:** Demographic characteristics of mothers, fathers, primary caregivers, and secondary caregivers.

	Mothers (*N* = 157)	Fathers (*N* = 113)	ANOVA or *χ*^2^	*p*	Primary caregivers (*N* = 147)	Secondary caregivers (*N* = 123)	ANOVA or χ^2^	*p*
Age, *M* (*SD*)	33.26 (3.99)	37.71 (5.95)	*F* (1, 263) = 53.142	<0.001	34.93 (5.19)	35.31 (5.56)	*F* (1, 263) = 0.336	0.563
Length of relationship (in years), *M* (*SD*)	7.24 (3.07)	9.32 (3.94)	*F* (1, 268) = 23.756	<0.001	7.98 (3.53)	8.27 (3.69)	*F* (1, 268) = 0.429	0.513
Relationship status: married, *n* (%)	87.3	69.9	*χ*^2^(1) = 12.362	<0.001	80.3	79.7	*χ*^2^(1) = 0.015	0.903
Infant is a twin, *n* (%)	7.0	25.7	*χ*^2^(1) = 18.124	<0.001	13.6	16.3	*χ*^2^(1) = 0.374	0.541
Working status, *n* (%)			*χ*^2^(2) = 8.089	<0.05			*χ*^2^(2) = 20.333	<0.001
Full time	56.1	69.6			51.7	73.8		
Part-time	31.2	16.1			27.2	22.1		
Not working	12.7	14.3			21.1	4.1		
Educational level, *n* (%)			*χ*^2^(2) = 7.226	<0.05			*χ*^2^(2) = 1.111	0.574
High	87.7	75.2			82.8	82.1		
Middle	11.0	21.2			15.9	14.6		
Low	1.3	3.5			1.4	3.3		
Family income, *n* (%)			*χ*^2^(2) = 7.626	< 0.05			*χ*^2^(2) = 0.431	0.806
Over 42,356 dollars	65.2	80.5			73.3	69.7		
Between 12,706–42,356 dollars	32.9	18.6			25.3	28.5		
Under 12,706 dollars	1.9	0.9			1.4	1.6		
Country of residence, *n* (%)			*χ*^2^(2) = 10.264	< 0.05			*χ*^2^(2) = 0.343	0.842
The Netherlands	42.7	23.9			36.1	33.3		
The United Kingdom	19.7	27.4			21.8	24.4		
France	37.6	48.7			42.2	42.3		
Living location, *n* (%)			*χ*^2^(3) = 7.092	0.069			*χ*^2^(3) = 0.544	0.909
Large city	22.9	37.2			29.3	28.5		
Medium city	33.8	29.2			33.3	30.1		
Small city	37.6	27.4			32.0	35.0		
Rural area	5.7	6.2			5.4	6.5		

### Procedure

The study obtained ethical approval from the collaborating research institutes in the Netherlands, the United Kingdom, and France. Participants were recruited in these three countries via online forums, magazines, surrogacy-lawyers, parent support groups, and fertility clinics (for more information about this procedure, see Rubio et al., 2017). To be included in the study, parents had to meet several inclusion criteria. All couples used artificial/assisted reproductive techniques to become parents for the first time of either singletons or twins. Same-sex male parents used egg donation and surrogate females, same-sex female parents used anonymous sperm donation for one of the mothers to become pregnant, and different-sex parents used IVF for the mother to become pregnant without sperm or egg donation. All infants were around 4 months old when the assessment took place.

After meeting the inclusion criteria and giving (informed) consent, parents were separately invited before the home-visit to fill in online standardized questionnaires about demographic characteristics and child temperament. When the infant was between 3.5 and 4.5 months old, the assessment took place in the parents’ home and additional online standardized questionnaires, audio-recorded standardized semi-structured interviews, and three video-recorded observations were conducted by trained researchers. For this study only several standardized questionnaires (about caregiving tasks, child temperament, and parenting stress) and the video-recorded observations during the home-visit were relevant.

Each parent was videorecorded interacting with the infant in three daily caregiving task contexts: cleaning, feeding, and playing. The other parent was not present during this observation. Both parents were separately observed cleaning, feeding, and playing at the time appropriate for the infant. In the cleaning context, the parent had to change the infants’ diaper or bathe the infant. Observations started when the infant was put on the changing mat and continued until the cleaning act was clearly finished, and the infant was removed from the changing mat. In the feeding context, the parent had to breastfeed or bottle feed the infant. Observations started when the food was presented to the infant, until the food was finished, or the infant would not eat anymore. In the playing context, the parent was asked to play with the infant as they normally did for 10 min.

### Measures

#### Observations of positive affect and negative affect during daily caregiving tasks

The three video-recorded observations of the parent and the infant in the cleaning, feeding, and playing contexts were used to measure the parents’ positive and negative affect toward their infant. At least two trained researchers from the parents’ country coded the video-recorded observations using coding scales for positive and negative affect.^1^ To ensure inter-rater reliability, the researchers discussed the coding until they came to consensus. To ensure maintenance of agreement across the three countries, 22% of the videos were re-coded by a coder from another country.

##### Positive affect

The amount and quality of positive parental affect was indexed by “(a) warm facial expressions (e.g., smiling) showing interest in the baby, (b) vocalizations with a happy or playful intonation, affectionate phrases and laughs, (c) affectionate touching, like kissing and stroking, and (d) playful, game-like interaction (see text footnote 1).” Positive affect was rated on a scale from 1 to 4. A score of 1 was given when the parent expressed little or no positive affect to the infant and had a neutral/negative face and voice. A score of 2 was given when the parent expressed positive affect of a forced/stiff quality or which was inappropriate to the interaction. A score of 3 was given when the parent expressed some positive affect to the infant which was natural, relaxed, and spontaneous. A score of 4 was given when the parent predominantly expressed positive affect, appropriate to the interaction, in a genuine and spontaneous way. A higher score indicated more positive affect than a lower score. Average absolute intraclass correlations indicated adequate inter-rater reliability between two coders, 0.80, 95% CI = 0.77, 0.82, and among three coders in 22% of the videos, 0.73, 95% CI = 0.66, 0.79.

##### Negative affect

Negative affect was measured as the frequency with which the parent directed a negatively toned facial or vocal expression toward the infant (see footnote 1). Negative affect was rated on a scale from 1 to 4. A score of 1 (*no negative affect*) was given when the parent showed no negative affect toward the infant. A score of 2 (*low negative affect*) was given when the parent expressed one instance of low-level negative affect. Low-level negative affect was indicated by impatience, irritation, resentment, rolling of the eyes, teasing, or adopting a long-suffering attitude. A score of 3 (*moderate negative affect*) was given when the parent expressed more than one instance of low-level negative affect. A score of 4 (*clear negative affect*) was given when the parent expressed at least one instance of clear anger or displeasure toward the infant. Overt anger or displeasure was seen as the highest level of negative affect and was indexed by speaking in a sharp, harsh, or raised voice, making negative remarks about the infant, or threatening the infant. For the parent behavior to be indexed as negative, the infant was not required to responded negatively. A higher score indicated more negative affect than a lower score. Average absolute intraclass correlations indicated adequate inter-rater reliability between two coders, 0.81, 95% CI = 0.78, 0.83, and among three coders in 22% of the videos, 0.70, 95% CI = 0.59, 0.78.

#### Control variables

##### Child temperament

To measure the temperament of the infant, the Infant Characteristics Questionnaire (ICQ) was used. There were English (Bates et al., 1979), French (Bertrais et al., 1999), and Dutch (Kohnstamm, 1984) versions. Only the primary caregiver filled in the questionnaire before the home visit and only the subscale “Fussiness/Difficulty,” consisting of six items, was used. The items measured the parents’ perception of their infants’ temperament by asking the parent to rate the difficult/fussiness of the infant on a scale from 1 (*easier behavior*) to 7 (*most problematic behavior*). An example item was: “*How easy or difficult it is for you to calm or soothe your baby when he/she is upset?*.” A mean score was used for the analyses, with a higher score indicating more fussiness/difficulty in the infants’ temperament and a lower score indicating an easy temperament. The Fussiness/Difficulty subscale had good internal consistency in this sample (*α* = 0.79).

##### Parenting stress

To measure parenting stress, the short version of the Parenting Stress Index (PSI) questionnaire was completed (Abidin, 2012) in the language of the parents. Both parents filled in the questionnaire during the home visit and only the subscale “Parental Distress,” consisting of 12 items, was used. An example item is: “*I feel trapped by my responsibilities as a parent*.” Parents answered the items on a scale from 1 (*strongly agree*) to 5 (*strongly disagree*). Scores ranged between 12 and 60, with a high score (score > 33) indicating high parenting distress. The Parental Distress subscale had good internal consistency in this sample (Cronbach’s *α* = 0.84).

### Data analytic approach

IBM SPSS Statistics version 29.0 was used for the statistical analyses. First, descriptive statistics (means, standard deviations, and correlations) were calculated. Then, the data were checked for outliers and the assumptions of normality, linearity, and homoscedasticity were checked. Multiple imputation was performed, with *m* = 20 imputations, to handle missing data (for more information about this procedure, see Van Rijn-van Gelderen et al., 2018, 2020; Ellis-Davies et al., 2022). As part of sensitivity analyses, we confirmed that the results were similar when the imputed data were not used. To investigate whether parental gender and caregiving role were associated with positive affect and negative affect in the feeding, cleaning, and playing contexts, six linear mixed models were conducted with parental gender (male/female), caregiving role (primary/secondary), and an interaction between parental gender and caregiving role as fixed effects. Contextual factors (singleton vs. twin status, country of residence) and parent–child factors (parenting stress [centered variable] and infant temperament [centered variable]), were added as covariates to control for their effects. In the models, family was added as a random effect to control for dependencies in the data. We checked whether we should control for different family types (different-sex parent families, same-sex male parent families, and same-sex female parent families) by running six linear mixed models (positive affect while feeding, cleaning, and playing, and negative affect while feeding, cleaning, and playing) with families as a random effect and family type as a parameter.

## Results

### Preliminary analyses

In Table 3 the descriptive statistics for the outcome variables (positive affect during feeding, cleaning, and playing, and negative affect during feeding, cleaning, and playing) and the continuous covariates (child temperament and parenting stress) are presented, including the number of missing values for which the multiple imputations were used. The correlations between the outcome variables and the continuous covariates are presented in Table 4.

**Table 3:** Means and standard errors for positive affect, negative affect, child temperament, and parenting stress by parental gender and caregiving role.

	Mothers (*N* = 157)		Fathers (*N* = 113)		Primary caregiver (*N* = 147)		Secondary caregiver (*N* = 123)		Total (*N* = 270)	
	*M*	*SE*	*M*	*SE*	*M*	*SE*	*M*	*SE*	*M*	*SE*
Positive affect during feeding ^a^	2.65	0.074	2.37	0.093	2.50	0.076	2.57	0.092	2.53	0.059
Positive affect during cleaning ^b^	2.99	0.058	2.92	0.070	2.99	0.057	2.93	0.070	2.96	0.044
Positive affect during playing ^c^	2.98	0.053	2.88	0.063	2.97	0.055	2.90	0.060	2.94	0.041
Negative affect during feeding ^d^	1.73	0.078	1.84	0.094	1.71	0.078	1.85	0.098	1.77	0.062
Negative affect during cleaning ^e^	1.84	0.074	1.78	0.081	1.75	0.072	1.88	0.082	1.81	0.054
Negative affect during playing ^f^	1.76	0.069	1.99	0.090	1.81	0.072	1.91	0.086	1.86	0.055
Child temperament: fussiness ^g^	3.067	0.061	2.766	0.063	2.928	0.060	2.957	0.068	2.941	0.045
Parenting stress ^h^	21.671	0.454	21.763	0.712	22.016	0.533	21.344	0.596	21.710	0.397

Calculated from the pooled dataset from the *m* = 20 imputations.

Number of missing values:

a *n* = 52 (19.26%).

b *n* = 8 (2.96%).

c *n* = 2 (0.74%).

d *n* = 52 (19.26%).

e *n* = 8 (2.96%).

f *n* = 3 (1.11%).

g *n* = 4 (1.48%).

h *n* = 2 (0.74%).

**Table 4:** Correlations among positive affect, negative affect, child temperament, and parenting stress.

	Positive affect – feeding	Positive affect – cleaning	Positive affect – playing	Negative affect – feeding	Negative affect – cleaning	Negative affect – playing	Child temperament	Parenting stress
Positive affect – feeding	1							
Positive affect – cleaning	0.147*	1						
Positive affect – playing	0.288**	0.279**	1					
Negative affect – feeding	−0.244**	−0.100	−0.180**	1				
Negative affect – cleaning	−0.151*	−0.254**	0.403**	0.369**	1			
Negative affect – playing	−0.126	−0.171**	−0.237**	0.358**	0.403**	1		
Child temperament	−0.013	0.077	−0.081	0.055	−0.116	−0.068	1	
Parenting stress	−0.089	−0.042	−0.139*	0.090	−0.073	0.127*	0.232**	1

Calculated from the pooled dataset from the *m* = 20 imputations.

*N* = 270.

*Significant with *p* = 0.05 as criterion for significance (two-tailed).

**Significant with *p* = 0.01 as criterion for significance (two-tailed).

Checking for outliers revealed univariate outliers for positive affect during cleaning and playing. Upon closer inspection of these outliers, it appeared that these outliers were values of 1 and 4, which are the end points on the scale. Since 1 and 4 are plausible values on these scales, it was decided on substantive grounds to not remove these outliers and not to conduct a sensitivity analysis.

The assumption of normality was checked using a histogram and normal probability plot of the residuals. The assumptions of linearity and homoscedasticity were also checked using a scatterplot of the residuals and predicted values. The assumptions were not met, due in part to the kind of data and the scale used for the outcome variables. The distribution of the residuals appeared bimodal instead of normal for the negative affect outcomes but transforming the data to achieve normality would have made the results less interpretable (Schielzeth et al., 2020). Because non-normality influences results minimally (Schielzeth et al., 2020), the data were not transformed.

The intraclass correlations in six linear models (for the six outcome variables) revealed a random effect of family that varied between 0.07 and 0.37, indicating that observations within one family were dependent. Because Musca et al. (2011) showed that even small intraclass correlations, such as 0.01, can cause Type I error rates to inflate family was added as a random effect to the models.

We found no differences between family types except for positive affect during feeding and negative affect during playing. For positive affect during feeding, same-sex female parent families showed more positive affect than different-sex parent families (Estimate = 0.322, SE = 0.154, *p* = 0.037, 95% CI [0.019; 0.626]). For negative affect during playing, same-sex male parent families showed less negative affect than different-sex parent families (Estimate = −0.306, SE = 0.155, *p* = 0.048, 95% CI [−0.610; −0.003]) and same-sex female parent families showed less negative affect than different-sex parent families (Estimate = −0.456, SE = 0.138, *p* < 0.001, 95% CI [−0.726; −0.185]). We therefore added family type as a covariate in the linear mixed models for positive affect during feeding and negative affect during playing to control for the effect.

### Positive affect

Table 5 shows the results of the three linear mixed models examining positive affect separately in each of the contexts. The results indicated that parental gender, parental caregiving role, and an interaction between parental gender and caregiving role did not predict parental positive affect significantly in any context. Similarly, child temperament, parenting stress, and singleton versus twin status were not significant predictors of parental positive affect in any context. Despite the significant differences found in the preliminary analyses between family type in positive affect during feeding, family type was not a significant predictor in this linear mixed model.

**Table 5:** Linear mixed models for positive affect during feeding, cleaning, and playing.

	Positive affect														
	Feeding					Cleaning					Playing				
Effect	Estimate	*SE*	95% CI		*p*	Estimate	*SE*	95% CI		*p*	Estimate	*SE*	95% CI		*p*
			Lower limit	Upper limit				Lower limit	Upper limit				Lower limit	Upper limit	
Fixed effects															
Intercept	2.571	0.198	2.180	2.962	<0.001	3.052	0.120	2.816	3.288	<0.001	3.195	0.101	2.996	3.394	<0.001
Parental gender ^a^	0.230	0.256	−0.272	0.733	0.368	0.079	0.135	−0.186	0.344	0.559	0.035	0.112	−0.184	0.254	0.755
Parental caregiving role ^b^	−0.336	0.182	−0.694	0.022	0.066	0.169	0.138	−0.102	0.440	0.221	0.041	0.108	−0.171	0.253	0.702
Parental gender * parental caregiving role	0.285	0.244	−0.193	0.764	0.242	−0.205	0.182	−0.561	0.152	0.260	0.016	0.145	−0.268	0.299	0.913
Child temperament	−0.032	0.078	−0.185	0.121	0.681	0.082	0.063	−0.041	0.205	0.191	−0.051	0.054	−0.158	0.055	0.346
Parenting stress	−0.003	0.009	−0.020	0.015	0.757	−0.002	0.007	−0.016	0.012	0.748	−0.007	0.006	−0.019	0.005	0.264
Having singletons or twins ^c^	−0.040	0.166	−0.366	0.286	0.810	−0.087	0.138	−0.358	0.184	0.527	0.190	0.120	−0.046	0.427	0.114
Country of residence: U.K. – the Netherlands ^d^	−0.096	0.177	−0.446	0.255	0.590	−0.155	0.125	−0.400	0.090	0.216	−0.346	0.111	−0.564	−0.128	0.002
Country of residence: France – the Netherlands ^e^	−0.475	0.134	−0.738	−0.212	<0.001	−0.243	0.104	−0.447	−0.038	0.020	−0.611	0.095	−0.796	−0.425	<0.001
Family type: same-sex male parents – different-sex parents	0.304	0.223	−0.136	0.743	0.174										
Family type: same-sex female parents – different-sex parents	0.117	0.171	−0.219	0.453	0.496										
Random effects															
Within families variance	0.044	0.064	−0.083	0.171	0.495	0.024	0.045	−0.064	0.111	0.596	0.071	0.033	0.007	0.136	0.031

Calculated from the pooled dataset from the *m* = 20 imputations.

a 0 = male, 1 = female.

b 0 = secondary caregiver, 1 = primary caregiver.

c 0 = singleton, 1 = twins.

d 0 = the Netherlands, 1 = U.K.

e 0 = the Netherlands, 1 = France.

However, whether the parents came from France or the Netherlands was significantly related to their positive affect in all contexts whereas whether the parents came from the U.K. or the Netherlands was only significantly related to their positive affect while playing. The average mean differences displayed in Table 5 indicate that parents from the Netherlands showed the most positive affect toward their infants, followed by those from the United Kingdom (only significant for playing), with French parents showing the least positive affect toward their infant in all contexts. The results were the same when the analyses were computed using the dataset without imputation (see Supplementary materials), except for parental positive affect during feeding. In the dataset without the imputation, caregiver role was a significant predictor for positive affect during feeding (B (*SE*) = −0.389 (0.192), *p* = 0.045). However, the small estimate and the varying significance, demonstrate the instability of the effect.

### Negative affect

Table 6 shows the results of the three linear mixed models examining negative affect during each of the three contexts. The results indicated that parental gender, parental caregiving role, an interaction between parental gender and caregiving role, child temperament, parenting stress, and singleton versus twin status did not significantly predict negative affect during feeding and playing. However, whether the parents came from France or the Netherlands was significantly related to their negative affectivity in the feeding and playing contexts. The average mean differences in Table 6 showed that French parents were more negative toward their infant than Dutch parents in both feeding and playing contexts. In addition, whether the couples involved same-sex male or different-sex parents was significantly related to negative affect during playing. The average mean differences showed that same-sex male parents were less negative toward their infant than different-sex parents in the context of play.

**Table 6:** Linear mixed models for negative affect during feeding, cleaning, and playing.

	Negative affect														
	Feeding					Cleaning					Playing				
Effect	Estimate	*SE*	95% CI		*p*	Estimate	*SE*	95% CI		*p*	Estimate	*SE*	95% CI		*p*
			Lower limit	Upper limit				Lower limit	Upper limit				Lower limit	Upper limit	
Fixed effects															
Intercept	1.548	0.157	1.239	1.857	<0.001	1.618	0.142	1.340	1.895	<0.001	1.905	0.163	1.586	2.223	<0.001
Parental gender ^a^	0.025	0.180	−0.329	0.379	0.891	0.312	0.159	0.001	0.623	0.049	−0.153	0.224	−0.592	0.285	0.493
Parental caregiving role ^b^	−0.146	0.167	−0.473	0.181	0.380	−0.022	0.158	−0.333	0.288	0.888	0.022	0.171	−0.313	0.358	0.895
Parental gender * parental caregiving role	−0.004	0.227	−0.450	0.442	0.986	−0.278	0.211	−0.691	0.135	0.187	−0.118	0.224	−0.557	0.321	0.598
Child temperament	0.035	0.079	−0.120	0.191	0.654	−0.170	0.075	−0.316	−0.023	0.024	−0.110	0.074	−0.256	0.036	0.140
Parenting stress	0.003	0.009	−0.015	0.021	0.738	−0.009	0.009	−0.025	0.008	0.319	0.014	0.008	−0.003	0.031	0.101
Having singletons or twins ^c^	0.189	0.168	−0.142	0.519	0.263	−0.047	0.164	−0.369	0.275	0.774	−0.091	0.166	−0.416	0.234	0.584
Country of residence: U.K. – the Netherlands ^d^	−0.006	0.180	−0.363	0.350	0.972	−0.199	0.150	−0.493	0.095	0.185	0.278	0.152	−0.019	0.576	0.066
Country of residence: France – the Netherlands ^e^	0.635	0.143	0.353	0.918	<0.001	0.416	0.128	0.166	0.666	0.001	0.595	0.128	0.344	0.847	<0.001
Family type: same-sex male parents – different-sex parents											−0.439	0.186	−0.804	−0.074	0.018
Family type: same-sex female parents – different-sex parents											−0.237	0.169	−0.568	0.095	0.162
Random effects															
Within families variance	0.061	0.070	−0.077	0.200	0.382	0.078	0.062	−0.043	0.199	0.209	0.076	0.061	−0.043	0.196	0.212

Calculated from the pooled dataset from the *m* = 20 imputations.

a 0 = male, 1 = female.

b 0 = secondary caregiver, 1 = primary caregiver.

c 0 = singleton, 1 = twins.

d 0 = the Netherlands, 1 = U.K.

e 0 = the Netherlands, 1 = France.

By contrast, parental gender, child temperament, and whether parents came from France or the Netherlands significantly predicted parental negative affect in the cleaning context. Parents showed less negative affect toward their infants in the cleaning context when the infant had a more difficult temperament while French parents expressed more negative emotions toward their infants than Dutch parents did. More importantly, mothers expressed more negative affect in the cleaning context than fathers did, although the average mean difference between mothers and fathers was only 0.312 (on a scale from 1 to 4). Furthermore, when data for both twins were included in the analyses (*N* = 312 instead of *N* = 270), parental gender, and infant temperament were no longer significant predictors of negative affect during cleaning (for parental gender: B (*SE*) = 0.255 (0.150), *p* = 0.090; for infant temperament: B (*SE*) = −0.070 (0.068), *p* = 0.300), but the country of residence remained a significant predictor (B (*SE*) = 0.426 (0.126), *p* < 0.001). In the dataset without imputation, parental gender was also no significant predictor of negative affect during cleaning. The remaining results were the same when the analyses were computed using the dataset without imputation (see Supplementary materials).

## Discussion

Parental positive affect and negative affect are relevant for developing children, especially during infancy (Bornstein, 2012), because they are related to the development of parent–child attachment relationships (Cox et al., 1992), as well as aspects of children’s social–emotional, academic, and behavioral functioning later in life (Alemany et al., 2013; Taraban and Shaw, 2018; Samdan et al., 2020; Pali et al., 2022). The goal of this study was to investigate whether parental gender and caregiving role were associated with mothers’ and fathers’ positive affect and negative affect while interacting with their 4-month-old first-born infants in feeding, cleaning, and playing contexts. In addition, we investigated whether contextual factors, namely singleton versus twin status and country of residence (the Netherlands, the U.K., and France), and parent–child factors, namely parenting stress, and infant temperament, were related to positive and negative parental affect. Overall, the results indicated that positive and negative parental affect in the three contexts were not related to the gender or caregiving role of the parents or the interaction between gender and caregiving role, nor to parenting stress, infant temperament, and singleton versus twin status. However, positive and negative parental affect were related to whether the parents came from the Netherlands, the U.K. or France. This study is one of very few to include samples of both different-sex and same-sex couples who conceived using ART and were all observed interacting with their infants in three different contexts in light of previous evidence that parents behave differently in different contexts (Leyendecker et al., 1997; Van Vliet et al., 2022).

Contrary to previous research indicating that mothers show more positive affect toward their infant than fathers do (Lamb et al., 1982; Hwang, 1986; Field et al., 1987; Brundin et al., 1988), and that primary caregivers, who are mainly mothers, show more positive affect than secondary caregivers (Field, 1978; Sun and Roopnarine, 1996; Laflamme et al., 2002), this study revealed no differences between mothers and fathers or between primary and secondary caregivers in the levels of positive affect displayed in feeding, cleaning, and playing contexts. Likewise, there were no differences between mothers and fathers or between primary and secondary caregivers in the levels of negative affect directed toward their infants in the three contexts, even though previous research suggested that mothers and fathers would differ (Deater-Deckard, 1996; Kwon et al., 2012).

The present results differed from those obtained in previous studies, perhaps because previous studies did not distinguish between the impact of gender and caregiving role (Brundin et al., 1988), did not investigate the caregiving role of the parents at all (Deater-Deckard, 1996; Kwon et al., 2012), confused caregiving role with employment status (Heath, 1976; Grych and Clark, 1999), or failed to include both secondary caregiver mothers and primary caregiver fathers in their samples (Field, 1978; Lamb et al., 1982; Hwang, 1986; Field et al., 1987; Sun and Roopnarine, 1996; Laflamme et al., 2002). The design of this study differed and thus provided an opportunity to investigate the extent to which parental gender and caregiving role separately contributed to parenting qualities (Carone and Lingiardi, 2022). It is also noteworthy that the context of parenting has changed, with increased paternal involvement in caregiving in many countries (Cabrera et al., 2000; Yeung et al., 2001; Huerta et al., 2014). In addition, same-sex parent couples might divide caregiving tasks more equally, which may affect the results. The group of different-sex parent families, who conceived through IVF, might be more similar to same-sex parent families than expected (Imrie and Golombok, 2020), perhaps dividing tasks more equally than expected.

Previously reported findings might also differ from those reported here because this study included same-sex male and same-sex female couples. Prior studies mostly involved traditional families with different-sex parents and mothers as the primary caregivers. Same-sex male and same-sex female couples are relatively understudied in parenting research although there is a growing body of evidence that parental sexual orientation does not adversely affect children’s adjustment (Lamb, 2012; Golombok, 2021) as once believed and is associated with more egalitarian attitudes (Sutfin et al., 2008; Bos and Sandfort, 2010; Goldberg et al., 2012). It is also possible that parents who conceive using ARTs have distinctively different attitudes to parenthood and behave differently as a result (Mazrekaj et al., 2022). It is noteworthy that a previous analysis of the same parents’ sensitivity and intrusiveness showed no differences associated with parental gender or sexual orientation (Ellis-Davies et al., 2022). Earlier studies might not have captured the diversity of today’s parents adequately.

As in previous analyses of data involving the same sample (Ellis-Davies et al., 2022), we found differences related to the countries where the parents lived. French parents showed less positive affect and more negative affect than Dutch parents in all contexts. British and Dutch parents only differed with respect to positive affect while playing, with British parents showing less positive affect than Dutch parents but more positive affect than French parents. The fact that Dutch parents displayed more positive affect than both French and British parents might be because the Netherlands is one of the most supportive and tolerant countries for same-sex parents (Takács et al., 2016). Social support and involved, responsive parenting are positively associated (Rhoad-Crogalis et al., 2020) so societal support might make the parenting context more relaxed and enjoyable for Dutch than for French and British parents, leading Dutch parents to show more positive affect toward their infants. Furthermore, because surrogacy was and remains forbidden and donor insemination was forbidden for same-sex female couples until 2022 in France, whereas altruistic surrogacy is legal in the Netherlands (González, 2019), it may have been harder for French same-sex (male) couples to conceive children. Although parenting stress was not related to positive affect and negative affect in this study, other sources of stress, for example related to same-sex couples being a minority (Meyer, 2003), might be experienced at a higher level in French same-sex male couples, possibly leading French parents to show less positive affect and more negative affect toward their children than Dutch parents. In contrast to France, altruistic surrogacy is permitted in the United Kingdom (González, 2019) and different surrogacy policies might explain the differences between British and French parents.

The differences between Dutch, British and French parents might also be due to cultural differences in parenting styles (Lansford, 2022). Ellis-Davies et al. (2022) also reported differences between Dutch, French, and British parents in sensitivity and intrusiveness. These cross-country differences in parenting qualities underline the fact that even western European countries, which are similar in many respects can still differ. Future research on parenting qualities should take possible differences like this into account.

Contrary to our expectations, parenting stress, infant temperament, and singleton versus twin status were not related to positive affect and negative affect. It is possible that different operationalizations of parenting stress or stigma resulted in the same-sex male and same-sex female parents not being completely honest (Meyer and Wilson, 2009) or that the levels of stress experienced by these parents were relatively modest. Similarly, most of the temperament ratings were around the middle of the scale, with few difficult temperaments identified. We also relied exclusively on the fussiness/difficulty subscale to assess temperamental difficulty; other components of infant temperament, such as reactivity and self-regulation (Rothbart et al., 2000) or adaptability and unpredictability (Bates et al., 1979), might influence parental behavior. Finally, few (14.8%) of the parents had twins, limiting our ability to recognize effects of this status and the fact that we observed the parents of twins in one-on-one interactions with their infants might have reduced the representativeness of the observed interactions.

Some limitations should also be noted. First, the fact that most parents in the sample were highly educated limits the generalizability of the results (Roubinov and Boyce, 2017). Besides that, parents’ gender was dichotomized in terms of males and females. However, it would have been a valuable addition to consider whether parents were cisgender or transgender. Future research might take that into account. In addition, we defined primary and secondary caregivers in each couple using proportion scores on “The Who Does What” questionnaire. This method of defining which parent is the primary and secondary caregiver might be less suitable for same-sex parents than for different-sex parents, because in same-sex parent couples caregiving tasks are known to be more equally divided. Moreover, the questionnaire solely included items about responsibility for caregiving tasks during the weekdays, which might fail to capture other primary caregiving tasks that matter as well, like attending healthcare appointments and emotional regulatory processes. Further, positive and negative affect were coded on a scale from 1 to 4 which may have been too restricted to adequately represent subtle differences. We also did not examine the children’s response to the parents’ displays of emotion although these might have affected the parents’ behavior. In addition, as discussed in the Results section, the assumptions for a linear mixed-model analysis were not met because the distribution of the residuals of the negative affect scores were bimodally distributed, suggesting that the dataset included two normal distributions (Schielzeth et al., 2020). According to Schielzeth et al. (2020), this kind of non-normality minimally influences results but the bimodal distribution might indicate that a (binary) predictor was missing from the model.

Notwithstanding these limitations, the study showed that neither gender nor caregiving role affected the levels of positive and negative affect directed by parents to their 4-month-old infants. Future research should investigate whether these and other aspects of parenting quality are affected by parental gender or caregiving role during childhood and adolescence as well.

## Data availability statement

The data analyzed in this study is subject to the following licenses/restrictions: controversial topic. Requests to access these datasets should be directed to L.vanRijn-vanGelderen@uva.nl.

## Ethics statement

The study obtained ethical approval from the collaborating research institutes in the Netherlands, the United Kingdom, and France. The studies were conducted in accordance with the local legislation and institutional requirements. The participants’ legal guardians/next of kin provided written informed consent.

## Author contributions

TL: Formal analysis, Writing – original draft, Writing – review & editing. KE-D: Investigation, Supervision, Writing – review & editing. BR: Investigation, Writing – review & editing. OV: Funding acquisition, Investigation, Writing – review & editing. HB: Funding acquisition, Supervision, Writing – review & editing, Investigation. ML: Funding acquisition, Supervision, Writing – review & editing. LV: Conceptualization, Investigation, Project administration, Supervision, Writing – review & editing.

## Footnotes

^1^The authors KE-D, LV, AW, OV, and BR were part of the coding team. They were trained to use a coding scheme that was developed by Nanmathi Manian, under the supervision of Marc Bornstein from the National Institute of Child Health and Development (NICHD) based upon the Emotional Availability Scales (Biringen et al., 2000). More information is available upon request.
