# Supplementary material for: Parental positive affect and negative affect in same- and different-sex parent families: no associations with parental gender and caregiving role
Source: Front Psychol. 2024 Mar 7;15:1332758. doi: 10.3389/fpsyg.2024.1332758 (PMC10956513; doi:10.3389/fpsyg.2024.1332758)
Supplement: Supplementary file 1 [file Data_Sheet_1.docx]

**Appendix A**

**Table A1**

*Linear Mixed Models for Positive Affect during Feeding, Cleaning, and Playing, Without the Imputed Data.*

|  | Positive affect | | | | | | | | | | | | | | |
| --- | --- | --- | --- | --- | --- | --- | --- | --- | --- | --- | --- | --- | --- | --- | --- |
|  | Feeding | | | | | Cleaning | | | | | Playing | | | | |
| Effect | Estimate | *SE* | 95% CI | | *p* | Estimate | *SE* | 95% CI | | *p* | Estimate | *SE* | 95% CI | | *p* |
|  |  |  | Lower limit | Upper limit |  |  |  | Lower limit | Upper limit |  |  |  | Lower limit | Upper limit |  |
| Fixed effects |  |  |  |  |  |  |  |  |  |  |  |  |  |  |  |
| Intercept | 2.519 | 0.194 | 2.136 | 2.902 | <.001 | 3.007 | 0.122 | 2.767 | 3.247 | <.001 | 3.205 | 0.104 | 3.000 | 3.409 | <.001 |
| Parental gender ^a^ | 0.347 | 0.261 | -0.170 | 0.863 | .186 | 0.123 | 0.136 | -0.144 | 0.391 | .363 | 0.038 | 0.113 | -0.184 | 0.261 | .735 |
| Parental caregiving role ^b^ | -0.389 | 0.192 | -0.768 | -0.009 | .045 | 0.214 | 0.139 | -0.060 | 0.489 | .125 | 0.061 | 0.110 | -0.156 | 0.278 | .580 |
| Parental gender * parental caregiving role | 0.259 | 0.255 | -0.244 | 0.762 | .311 | -0.252 | 0.182 | -0.611 | 0.107 | .169 | -0.007 | 0.146 | -0.295 | 0.281 | .962 |
| Child temperament | -0.037 | 0.080 | -0.194 | 0.120 | .644 | 0.082 | 0.062 | -0.041 | 0.205 | .192 | -0.052 | 0.054 | -0.159 | 0.054 | .334 |
| Parenting stress | -0.002 | 0.009 | -0.020 | 0.016 | .804 | -0.002 | 0.007 | -0.016 | 0.013 | .815 | -0.006 | 0.006 | -0.018 | 0.006 | .324 |
| Having singletons or twins ^c^ | -0.087 | 0.185 | -0.453 | 0.279 | .640 | -0.064 | 0.138 | -0.337 | 0.210 | .646 | 0.205 | 0.123 | -0.038 | 0.449 | .098 |
| Country of residence: U.K. – the Netherlands ^d^ | -0.101 | 0.188 | -0.471 | 0.270 | .593 | -0.169 | 0.127 | -0.420 | 0.082 | .185 | -0.384 | 0.112 | -0.607 | -0.162 | <.001 |
| Country of residence: France – the Netherlands ^e^ | -0.479 | 0.137 | -0.751 | -0.207 | <.001 | -0.234 | 0.106 | -0.445 | -0.024 | .029 | -0.628 | 0.097 | -0.820 | -0.436 | <.001 |
| Family type: same-sex male parents – different-sex parents | 0.400 | 0.220 | -0.035 | 0.834 | .071 |  |  |  |  |  |  |  |  |  |  |
| Family type: same-sex female parents – different-sex parents | 0.126 | 0.173 | -0.214 | 0.467 | .466 |  |  |  |  |  |  |  |  |  |  |
| Random effects |  |  |  |  |  |  |  |  |  |  |  |  |  |  |  |
| Within families variance | 0.065 | 0.071 | 0.008 | 0.558 | .361 | 0.023 | 0.046 | 0.000 | 1.157 | .616 | 0.074 | 0.033 | 0.031 | 0.178 | .026 |

Note. ^a^ 0 = male, 1 = female

^b^ 0 = secondary caregiver, 1 = primary caregiver

^c^ 0 = singleton, 1 = twins

^d^ 0 = the Netherlands, 1 = U.K.

^e^ 0 = the Netherlands, 1 = France

**Table A2**

*Linear Mixed Models for Negative Affect during Feeding, Cleaning, and Playing, Without the Imputed Data.*

|  | Negative affect | | | | | | | | | | | | | | | | |  |
| --- | --- | --- | --- | --- | --- | --- | --- | --- | --- | --- | --- | --- | --- | --- | --- | --- | --- | --- |
|  | Feeding | | | | | Cleaning | | | | | | Playing | | | | | |  |
| Effect | Estimate | *SE* | 95% CI | | *p* | | Estimate | *SE* | 95% CI | | *p* | | Estimate | *SE* | 95% CI | | *p* | |
|  |  |  | Lower limit | Upper limit |  | |  |  | Lower limit | Upper limit |  | |  |  | Lower limit | Upper limit |  | |
| Fixed effects |  |  |  |  |  | |  |  |  |  |  | |  |  |  |  |  | |
| Intercept | 1.535 | 0.165 | 1.210 | 1.860 | <.001 | | 1.626 | 0.147 | 1.335 | 1.916 | <.001 | | 1.952 | 0.166 | 1.624 | 2.280 | <.001 | |
| Parental gender ^a^ | 0.088 | 0.196 | -0.299 | 0.474 | .654 | | 0.299 | 0.162 | -0.021 | 0.619 | .067 | | -0.176 | 0.227 | -0.624 | 0.272 | .439 | |
| Parental caregiving role ^b^ | -0.183 | 0.175 | -0.529 | 0.164 | .299 | | -0.051 | 0.165 | -0.375 | 0.274 | .759 | | 0.010 | 0.176 | -0.338 | 0.357 | .957 | |
| Parental gender * parental caregiving role | -0.025 | 0.238 | -0.495 | 0.444 | .915 | | -0.250 | 0.216 | -0.675 | 0.176 | .248 | | -0.108 | 0.228 | -0.559 | 0.342 | .636 | |
| Child temperament | 0.034 | 0.081 | -0.125 | 0.193 | .676 | | -0.172 | 0.076 | -0.322 | -0.023 | .024 | | -0.110 | 0.075 | -0.257 | 0.037 | .143 | |
| Parenting stress | 0.002 | 0.009 | -0.016 | 0.021 | .804 | | -0.008 | 0.009 | -0.025 | 0.009 | .358 | | 0.014 | 0.009 | -0.003 | 0.031 | .095 | |
| Having singletons or twins ^c^ | 0.291 | 0.184 | -0.074 | 0.656 | .117 | | -0.054 | 0.171 | -0.392 | 0.283 | .751 | | -0.086 | 0.168 | -0.419 | 0.247 | .611 | |
| Country of residence: U.K. – the Netherlands ^d^ | 0.000 | 0.187 | -0.370 | 0.370 | .999 | | -0.169 | 0.157 | -0.479 | 0.140 | .281 | | 0.252 | 0.154 | -0.052 | 0.556 | .104 | |
| Country of residence: France – the Netherlands ^e^ | 0.628 | 0.139 | 0.354 | 0.902 | <.001 | | 0.422 | 0.131 | 0.162 | 0.682 | .002 | | 0.560 | 0.131 | 0.301 | 0.819 | <.001 | |
| Family type: same-sex male parents – different-sex parents |  |  |  |  |  | |  |  |  |  |  | | -0.422 | 0.190 | -0.796 | -0.048 | .027 | |
| Family type: same-sex female parents – different-sex parents |  |  |  |  |  | |  |  |  |  |  | | -0.245 | 0.169 | -0.578 | 0.089 | .150 | |
| Random effects |  |  |  |  |  | |  |  |  |  |  | |  |  |  |  |  | |
| Within families variance | 0.099 | 0.075 | 0.023 | 0.433 | .184 | | 0.079 | 0.065 | 0.016 | 0.399 | .226 | | 0.069 | 0.061 | 0.012 | 0.395 | .261 | |

Note. ^a^ 0 = male, 1 = female

^b^ 0 = secondary caregiver, 1 = primary caregiver

^c^ 0 = singleton, 1 = twins

^d^ 0 = the Netherlands, 1 = U.K.

^e^ 0 = the Netherlands, 1 = France
